# Significant Accumulation of Iodine and Selenium in Chicory (*Cichorium intybus* L. var. *foliosum* Hegi) Leaves after Foliar Spraying

**DOI:** 10.3390/plants9121766

**Published:** 2020-12-13

**Authors:** Mateja Germ, Nina Kacjan-Maršić, Ana Kroflič, Ana Jerše, Vekoslava Stibilj, Aleksandra Golob

**Affiliations:** 1 Biotechnical Faculty, University of Ljubljana, 1501 Ljubljana, Slovenia; mateja.germ@bf.uni-lj.si (M.G.); nina.kacjan.marsic@bf.uni-lj.si (N.K.-M.); 2 Jožef Stefan Institute, 1501 Ljubljana, Slovenia; ana.kroflic@gmail.com (A.K.); ana.jerse@gmail.com (A.J.); vekoslava.stibilj@gmail.com (V.S.)

**Keywords:** agronomy, biofortification, mineral nutrition, crop

## Abstract

The interactions between the uptake of selenium (as selenite and selenate) and iodine (as iodate and iodide) by red chicory (*Cichorium intybus* L. var. *foliosum* Hegi) and their effects on selected morphological and physiological characteristics were investigated. Seedlings were transplanted to the field, and at the onset of head formation, the plants were foliar-sprayed with the following solutions: Milli-Q water (control), Se (IV), Se (VI), I (−I), I (V), Se (IV) + I (−I), Se (IV) + I (V), Se (VI) + I (−I) and Se (VI) + I (V). The different treatments had no significant effects on the yield (39.8–51.5 t ha^−1^) and mass (970–1200 g) of the chicory heads. The selenium content in Se-treated plants was up to 5.5-times greater than the control plants. The iodine content in the chicory leaves enriched with I was 3.5-times greater than the control plants. Iodide or iodate, applied together with selenite in the spray solution, increased the uptake of Se by chicory plants, while both forms of iodine, applied together with selenate, reduced the uptake of Se. Plants treated with I (V) had lower amounts of chlorophyll *a* and carotenoids than the control, while respiratory potential was higher than the control, which indicated the possible presence of stress in I (V)-treated plants. However, the potential photochemical efficiency of photosystem II was similar and close to the theoretical maximum (0.83) in the control and treated groups, which indicated that all of the plants were in good condition. Furthermore, the plant mass and yield were comparable in the control and treated groups. Molecular studies, like gene expression analysis, would represent a major upgrade of the present study by defining the mechanisms of Se and I uptake and their interactions and by enhancing the knowledge of the Se and I transporters.

## 1. Introduction

The trace elements iodine (I) and selenium (Se) are essential for the normal function of the thyroid gland [1]. The simultaneous biofortification of crops with I and Se is suitable in areas where there is a deficiency of both elements. The main reason for insufficient intake of I and Se in humans is their low content in vegetables [2,3]. Consequently, their uptake and further transfer along the food chain is low.

Iodine is a micronutrient that is essential for the correct physiological functioning of humans and animals (mainly mammals) [4]. The World Health Organization has identified I deficiency as one of the main factors that affect human health [5]. Iodine-deficiency disorders are the consequence of insufficient secretion of thyroid hormones, the obvious sign of which is goitre, the enlargement of the thyroid gland [6]. All age groups are at risk, and severe I deficiency can result in foetal damage, perinatal and infant mortality, endemic goitre, irreversible mental retardation and brain damage [6].

Plants absorb I in the form of iodide or iodate. Iodine, as the iodide anion, reaches the cells via the chloride transport pathway [7]. Iodine is not essential for plants, although more and more studies have shown that it is involved in plant physiological and biochemical processes [8]. It was recently discovered that, in spinach plants, I uptake is predominantly passive, but I (−I) can be absorbed actively through the symplast. Spinach leaves can absorb I via foliar fertilization, but its translocation is strongly limited [9].

Selenium is important for the normal function of a number of Se-dependent antioxidant enzymes, such as glutathione peroxidase and thioredoxin reductase [10]. Selenium has important biological functions, which range from protection against cancer to an influence on hormone metabolism [11]. Many epidemiological studies have confirmed that Se deficiency in the diet increases the incidence of cardiovascular disease, leads to thyroid dysfunction, and impairs the function of the immune and nervous systems [12]. A lack of Se has been linked to the occurrence of Kaschin-Beck disease, Keshan disease and chronic diseases associated with oxidative damage [13].

Plants absorb Se in the form of selenate or selenite. Selenate is actively transported by sulphate transporters [14], while selenite is assimilated via phosphate transporters [15]. Selenium essentiality for plants is still under discussion. However, there is increasing experimental evidence of a protective role of Se in plants [16] as an antioxidant and growth-promoting agent. Iodine and Se can stimulate plant growth at low concentrations, whereas they reduce it at higher concentrations. The threshold value depends on many factors, such as the plant species, developmental stage of the plant, technique of element addition, cultivation technique and others [3,11,16].

One of the first studies in which both elements were applied to plants simultaneously was published in 2004, where hydroponically grown spinach was enriched with Se and I [17]. In recent years, double enrichment has been performed for lettuce, carrot, pea sprouts and adult plants, *Brassica juncea*, buckwheat microgreens and adult plants and kohlrabi [3,18,19,20,21,22,23,24].

Chicory (*Cichorium intybus* L. var. *foliosum* Hegi) is a perennial herb that is used as forage for livestock, as a ‘folklore’ remedy and as a vegetable addition to the human diet [25]. It is a typical Mediterranean plant that is indigenous to Europe, Western Asia and North America, and shows great resistance to low temperatures [26]. In 2012, the production of chicory heads in Slovenia was 2562 tons, making it the third most produced leafy vegetable [27]. Annual chicory production in Slovenia in recent years has been estimated at 3002 tons [27,28]. Red chicory is known as a vegetable with high antioxidant activity. The presence of water-soluble flavonoids and other antioxidants has many positive effects on human health, such as protection against cancer, cardiovascular diseases and aging, among others [26,29].

The aim of the present study was to evaluate: (a) The accumulation by red chicory plants of I and Se; (b) the effects of I on Se contents in chicory leaves and vice versa; and (c) the effects of I and Se foliar application on selected morphological, biochemical and physiological parameters of red chicory plants.

## 2. Results and Discussion

### 2.1. Yield of Red Chicory

In the field experiments, chicory was sprayed with the following solutions: Sodium selenite (Na_2_SeO_3_)—Se (IV); sodium selenate (Na_2_SeO_4_)—Se (VI); potassium iodide (KI)—I (−I); potassium iodate (KIO_3_)—I (V); sodium selenite and potassium iodide—Se (IV) + I (−I); sodium selenite and potassium iodate—Se (IV) + I (V); sodium selenate and potassium iodide—Se (VI) + I (−I); or sodium selenate and potassium iodate—Se (VI) + I (V).

The foliar application with Se and I had no significant effects on the yield of chicory heads. These data showed that treatment with Se (VI) + I (−I) (43.9 t ha^−1^) and Se (VI) + I (V) (39.8 t ha^−1^) tended to reduce the chicory head the yield compared to the control (48.4 t ha^−1^). In addition, the treatments with I (−I) (40.1 t ha^−1^) and I (V) (43.5 t ha^−1^) also tended to reduce the yield compared to the control. The number of leaves that were removed to obtain the marketable mass of chicory heads, the marketable mass of chicory and the mass of the above ground plant parts were not different between the control and treated plants (Table 1). These data are generally in accordance with the findings of Zhu et al. [17], who studied the uptake of Se and I by spinach. They showed that there were no significant effects on plant shoot biomass with Se addition, while the addition of I in the substrate tended to reduce plants shoot biomass. On the other hand, Blasco et al. [30] reported that addition of I (−I) decreased the biomass of lettuce shoots, while the addition of I (V) increased the biomass of the edible parts of lettuce. For buckwheat, kohlrabi and pumpkins, it has been reported that foliar treatments with Se (IV), Se (VI), I (−I), I (V) and their combinations had no effects on their yield or biomass [23,24,31].

### 2.2. Selenium Content

Foliar application of Se (IV) or Se (VI) significantly increased the Se content in the chicory leaves compared to the control. Chicory leaves treated with Se (VI) had a higher Se content than those treated with Se (IV). Higher accumulation of Se after selenate spraying compared to selenite spraying has been reported for a number of crops, such as basil, spinach and buckwheat [23,32,33]. These differences in plant Se uptake and accumulation might be the consequence of different selenite and selenate uptake mechanisms [34] and might also be due to genetic differences [32]. It is known that selenite uptake in roots is through phosphorous transporters, while selenate uptake is through sulphur transporters [15]. However, there appear to be no data on the uptake mechanisms for Se through leaves.

**Figure 1:**
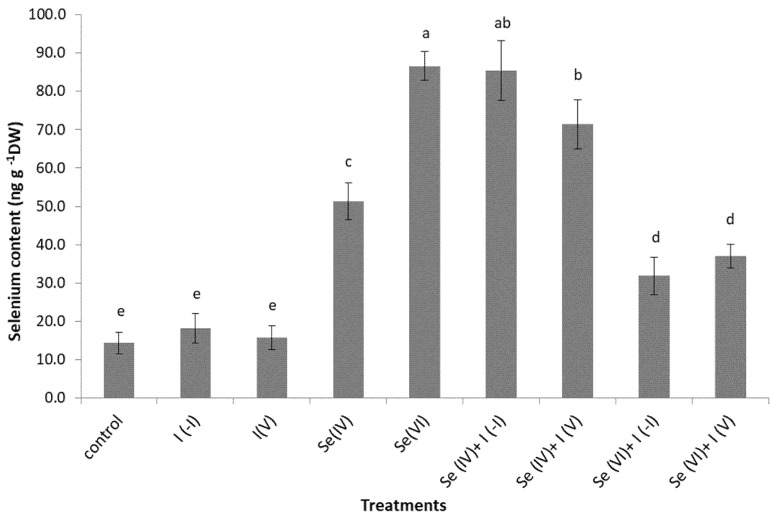
Selenium content in chicory leaves following the indicated foliar spraying. Data are means ± SE. Means followed by different superscript letters are significantly different at *p* < 0.05 (*n* = 5).

On the other hand, I showed variable impacts on Se accumulation in these Se-treated and I-treated chicory plants. Both forms of I increased Se levels in chicory heads when Se was added in the form of Se (IV), while both forms of I decreased Se levels when Se was added in the form of Se (VI) (Figure 1). A similar result was reported for common buckwheat seeds when exposed to the same treatments and the same concentrations as in the present study [23]. Selenium content in buckwheat seeds was lower when Se (VI) was applied together with I(V) or I(−I) compared to treatment with Se (VI) only, while I (V) significantly increased Se content in seeds when applied together with Se (IV) compared to treatment with Se(IV) only. In pumpkins, both forms of I increased Se accumulation in seeds when Se was added in the form of Se (VI) only [31]. On the other hand, I did not affect Se content in kohlrabi leaves and tubers [24]. The results regarding the effects of I to Se accumulation in different plant species are inconsistent, even when the same technique (i.e., foliar spraying) and the same concentrations of these elements are used for fertilization [3,23,24,31]. The observations are even more discrepant when different growth techniques and fertilization practises are compared. For example, positive effects of I on Se uptake have been shown for lettuce grown in an Nutrient Film Technique (NFT) hydroponic system [20], and negative effects of I on Se uptake have been reported for lettuce after soil fertilization with these two elements [35]. Negative effects of I on Se uptake have also been reported for carrot grown in soil fertilized with I and Se simultaneously [19].

### 2.3. Iodine Content

**Figure 2:**
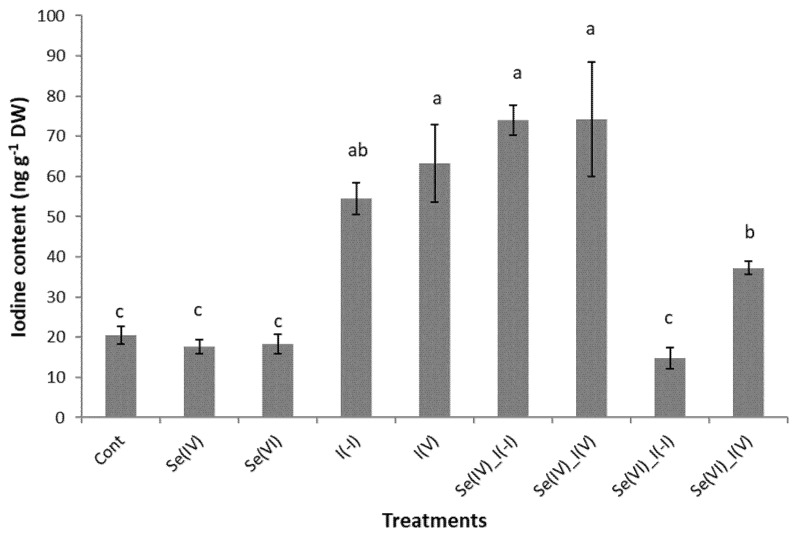
Iodine content in chicory leaves following the indicated foliar spraying. Data are means ± SE. Means followed by different superscript letters are significantly different at *p* < 0.05 (*n* = 5).

Foliar application of I(−I) or I(V) significantly increased I content in chicory leaves compared to the control (Figure 2). There were no significant differences between the I content of chicory leaves between the treatments with I(−I) and I(V). However, higher uptake of I (V) compared to I (−I) was reported for kohlrabi plants [24] and buckwheat microgreens [23]. Conversely, higher uptake of I(−I) compared to I (V) was reported for peas [3]. This is at least partly due to the different preferences of individual plant species regarding the uptake of iodide or iodate ions.

Selenite had no effects on I content in Se (IV) + I (−I)- and Se (IV) + I (V)-treated chicory, while Se (VI) reduced I content in Se (VI) + I (−I)- and Se (VI) + I (V)-treated chicory compared to the plants treated with I only (Figure 2). These data are consistent with those from a study by Smoleń et al. [20] for lettuce, in which lower uptake of I (V) in the presence of Se (VI) was reported.

Both forms of I lowered Se content when Se was added in the form of Se (VI), and the same form of Se lowered the amount of I when added simultaneously (Figure 1 and Figure 2). It appears that selenate and I ions competed for Se and I uptake in plant tissues. On the other hand, I ions stimulated the uptake or accumulation of selenite in chicory. These findings must be taken into account when fertilizing with Se and I at the same time to achieve the optimum content of both of these elements in chicory for human and animal nutrition.

For better understanding of the mechanisms for Se and I uptake and the interaction between Se and I in uptake through the leaves, measurements of molecular parameters, such as gene expression analysis, are needed.

### 2.4. Biochemical and Physiological Plant Parameters

Selected biochemical and physiological parameters of the chicory plants under the different treatments are presented in Table 2.

Plants treated with I(V) had slightly lower amounts of chlorophyll *a* and carotenoids than the control. On the other hand, the respiratory potential of these plants was higher than the control, which indicated increased metabolic activity of chicory plants treated with I (V). I (V)-treated plants also had higher ETS activity than the control in pea sprouts and adult pea plants [3,21], and in buckwheat seeds [23] treated with the same concentration of I (V), as in the present study. It is possible that, with increased metabolic activity, plants treated with I (V) protected themselves from the slight stress induced by this treatment. When organisms are under stress, they require more energy. Therefore, ATP production and O_2_ consumption in the mitochondria increase [36,37,38]. This is probably the reason why the treatment with I (V) did not affect the potential photochemical efficiency of photosystem II. On the other hand, Smoleń et al. [20] reported that, regardless of the cultivation type, such as field, soilless, or hydroponic, the iodide form I (-I) was more rapidly taken up by roots and that, at the same time, it was more toxic to the plants than the iodate form I (V) [39]. The potential photochemical efficiency of photosystem II was similar in the control and treated groups and was close to the theoretical maximum (0.83) [40]. This indicates that the plants were in good condition, which was also confirmed by the high yields here compared to those in other studies, i.e., 30 t ha^−1^ [41,42]. As there were no significant differences between the combinations in terms of the dry matter, yield, and photochemical efficiency of photosystem II, this shows that the application of I, Se and their combination did not have any negative impacts on these chicory plants. Our findings can be used to develop agronomic regulations regarding the simultaneous addition of I and Se to vegetables.

## 3. Materials and Methods

### 3.1. Plant Samples

The experiment was performed in the laboratory field of the Biotechnical Faculty, University of Ljubljana, in the central part of Slovenia (298 m above sea level, 46°35′ N, 14°55′ E). Seeds of red chicory cv. Leonardo (Austrosaat, Austria) were sown in June in polystyrene plug trays with 84 cells (cell volume: 35 mL) filled with peat substrate (Klassmann Neuhaus N3). The soil of the experimental site was classified as gleyic fluvisol and endogenic fluvisol and contained 26 g kg^−1^ soil organic matter in the 0–0.3 m soil layer. The average initial soil nitrate content was 6.8 mg kg^−1^ for the same depth, and the soil assimilable P and K were 24 mg kg^−1^ and 26 mg kg^−1^, respectively, on the basis of which the application rates of macronutrients were calculated according to the Regulations on Integrated Production of Vegetables [43]. One day before transplanting, granulated mineral fertilizers were incorporated into the plots at a rate of 70 kg N ha^−1^, 30 kg *p* ha^−1^ and 130 kg K ha^−1^ and 20 kg Ca ha^−1,^ as calcium ammonium-nitrate, super phosphate and potassium sulphate, respectively. The remaining N (70 kg ha^−1^) was applied 6 weeks after the plants were transplanted.

In July, seedlings with 4 fully developed leaves were transplanted to the bed at a planting density of 0.35 m × 0.35 m (planting density of 66,667 plants ha^−1^). The experiment was laid out in 5 randomized replicates for each treatment and 4 plants were used per replication. The plants were foliar-sprayed with the following solutions: Milli-Q water (control), 10 mg L^−1^ Se (selenite and selenate), 1000 mg L^−1^ I (iodide, iodate) and the combinations of these at the onset of head formation (42 days after transplantation). The weather conditions during the experiment are presented in the supplementary materials (Table S1). Chicory heads were sampled 98 days after transplantation when the plants formed firm ball-shaped heads. The aboveground plant parts and trimmed plants (marketable mass of chicory plants) were weighed and the number of leaves removed was counted. Yield was determined by weighing the fresh marketable heads, multiplied by the number of plants per square meter, calculated based on the inter- and intra-row spaces. Yield was expressed in tons per hectare. Also, 20% of the total yield was deducted to take into account the tractor wheel paths where plants would not have been planted under normal field production technology.

Biochemical and physiological analyses for the potential photochemical efficiency of PSII and chloroplast pigments were performed on fresh plant material. For analysis of the Se and I content, the samples were dry-frozen (1–16 LSC, Christ Gamma), homogenized, milled (Pulverisette 7, FRITISCH, Idar-Oberstein, Germany) and stored at −20 °C until analysis.

### 3.2. Determination of Selenium and Iodine Contents

For the determination of Se content, 0.25 g lyophilized and milled chicory plants was placed into a microwave oven (Ultrawave, Milestone, VA, USA) in 4 mL HNO_3_ (s.p., Merck). Digestions were performed with the following program: 20 min ramp to 220 °C, and 15 min hold at 220 °C. Solutions were cooled to room temperature, diluted, and the Se content was measured in parallel using inductively coupled plasma–tandem mass spectrometry (ICP-QQQ, Agilent Technologies, Tokyo, Japan). The detailed procedure has been described elsewhere [21]. Standard reference material NIST 1570a (spinach leaves) was used to check the accuracy and precision of the measurements. Good agreement between the determined (121 ± 17) ng Se g^−1^ and certified (117 ± 8) ng Se g^−1^ values was obtained.

To determine I content, approximately 0.15 g lyophilized and milled chicory plants was weighed into glass vessels. For extraction, 10 mL Milli-Q water and 2 mL 25% tetramethylammonium hydroxide were added. Extracted samples were filtered and diluted to an appropriate concentration of tetramethylammonium hydroxide for analysis with ICP-QQQ. Again, a detailed description of the procedure can be found elsewhere [21]. The accuracy and precision of the results were checked with reference material BCR 129 (hay powder) and NIST 1573a (tomato leaves). The values obtained of 0.156 ± 0.006 μg g^−1^ and 0.81 ± 0.02 μg g^−1^, respectively, were in good agreement with the certified values for I in BCR 129, 0.167 ± 0.024 μg g^−1^, and with the informative value for I in NIST 1573a, 0.85 μg g^−1^.

### 3.3. Determination of Biochemical and Physiological Parameters

The chloroplast pigments (carotenoids, chlorophyll *a*, chlorophyll *b*) were determined according to the literature [44,45]. A weighed piece of fresh leaf was homogenized in a mortar and extracted in 10 mL acetone (100%). Absorbances of extracts were measured at 662 nm, 645 nm and 470 nm with a UV-VIS spectrometer (Lambda 12; PerkinElmer, Norwalk, CT, USA). The pigments content was calculated according to the method described by the authors of [44,45] and was expressed per gram dry weight. For this reason, we separately measured the fresh weight of 10 similar pieces of leaves for each treatment and then dried these in an oven at 105 °C to constant weight. We then calculated the dry weight to fresh weight ratio. The potential photochemical efficiency of photosystem (PSII), expressed as Fv/Fm, was measured using a portable fluorometer (PAM 2500 Portable Chlorophyll Fluorometer, WALZ). The fluorescence was excited with a saturation beam of “white light” (PPFD = 8000 µ^2^s, 0.8 s). In addition to the potential photochemical efficiency, the effective quantum yield of PSII was measured using a standard 60° angle clip and a saturation pulse of “white light” (PPFD = 9000 µ^2^s, 0.8 s). The activity of the terminal electron transport system (ETS) of the mitochondria was determined to estimate the respiratory potential [46,47]. The ETS activity was calculated as the reduction rate of the artificial electron acceptor INT, measured according to the absorbance of the reaction mixture at 490 nm against the blank within 10 min of termination of the reaction. The detailed procedure for this method has been described elsewhere [21].

### 3.4. Statistical Analysis

The Statgraphics Centurion XVI program (Statgraphics, Herdon, VA, USA) was used for the statistical analysis of the results. One-way analysis of variance (ANOVA) was performed to determine the significance of the effects of treatments on the morphological, biochemical and physiological parameters of the chicory plants. The differences between the treatments were estimated using Tukey’s post-hoc tests (HSD), with significance accepted at *p* = 0.05.

## 4. Conclusions

The following conclusion can be drawn from the present study:

- Selenium content increased in the Se and Se plus I foliar-treated plants.
- Iodine content increased in chicory plants treated with I and I plus Se, except for the of selenate and iodide combination.
- Treatment with Se and I is promising way to increase their concentrations in crops, and consequently in humans, without reducing yields. Both forms of I lowered Se content when Se was added together with I in the form of Se (VI), and the same form of Se lowered the amount of I when both were added simultaneously.
- The potential photochemical efficiency of PSII showed the good vitality of the plants.
- Different I and Se treatments did not have any significant effects on the yield and mass of the chicory heads.

## Figures and Tables

**Table 1:** Effects of different treatments on morphological characteristics of chicory heads.

Treatment	Yield of Chicory (t/ha)	Mass of Above-Ground Plant Parts (g)	Number of Removed Leaves	Marketable Mass of Chicory Heads (g)
Control	48.4 ± 4.0 ^a^	1148 ± 102 ^a^	17 ± 2 ^a^	726 ± 59 ^a^
I (−I)	40.1 ± 2.0 ^a^	1009 ± 49 ^a^	20 ± 2 ^a^	602 ± 30 ^a^
I (V)	43.5 ± 4.1 ^a^	1043 ± 86 ^a^	19 ± 1 ^a^	653 ± 62 ^a^
Se (IV)	51.5 ± 1.4 ^a^	1199 ± 37 ^a^	19 ± 1 ^a^	766 ± 22 ^a^
Se (VI)	46.9 ± 4.3 ^a^	1079 ± 68 ^a^	17 ± 1 ^a^	704 ± 64 ^a^
Se (IV) + I (−I)	50.1 ± 4.0 ^a^	1176 ± 87 ^a^	18 ± 2 ^a^	752 ± 60 ^a^
Se (IV) + I (V)	50.5 ± 3.8 ^a^	1174 ± 87 ^a^	17 ± 1 ^a^	758 ± 57 ^a^
Se (VI) + I (−I)	43.9 ± 1.9 ^a^	1097 ± 69 ^a^	19 ± 1 ^a^	659 ± 29 ^a^
Se (VI) + I (V)	39.8 ± 5.7 ^a^	970 ± 125 ^a^	16 ± 2 ^a^	597 ± 85 ^a^

**Legend:** Se (IV), sodium selenite (Na_2_SeO_3_); Se (VI), sodium selenate (Na_2_SeO_4_); I (−I), potassium iodide (KI); I (V), potassium iodate (KIO_3_). Data are the mean ± SE. Means followed by different superscript letters are significantly different at *p* < 0.05 (*n* = 5).

**Table 2:** Effects of different treatments on the selected biochemical and physiological parameters.

Treatment	Chlorophyll *a* (mg g^−1^ DW)	Chlorophyll *b* (mg g^−1^ DW)	Carotenoids (mg g^−1^ DW)	ETS Activity (μL O_2_ mg^−1^ DW h)	Fv/Fm
Control	16.6 ± 1.3 ^ab^	13.1 ± 1.1 ^ab^	4.4 ± 0.3 ^ab^	16.4 ± 0.9 ^de^	0.74 ± 0.02 ^a^
I (−I)	15.1 ± 2.2 ^abc^	9.32 ± 1.4 ^cde^	4.1 ± 0.6 ^abc^	16.5 ± 0.4 ^de^	0.74 ± 0.01 ^a^
I (V)	12.7 ± 1.2 ^c^	6.90 ± 0.8 ^e^	3.3 ± 0.2 ^c^	18.7 ± 0.7 ^abc^	0.75 ± 0.01 ^a^
Se (IV)	14.1 ± 1.5 ^bc^	11.1 ± 1.3 ^bcd^	3.5 ± 0.3 ^bc^	16.9 ± 0.8 ^cde^	0.76 ± 0.01 ^a^
Se (VI)	18.9 ± 0.8 ^a^	14.4 ± 0.9 ^a^	5.0 ± 0.2 ^a^	20.2 ± 0.7 ^ab^	0.77 ± 0.02 ^a^
Se (IV) + I (−I)	15.0 ± 2.3 ^abc^	9.04 ± 1.4 ^cde^	4.1 ± 0.5 ^abc^	19.4 ± 1.5 ^ab^	0.77 ± 0.01 ^a^
Se (IV) + I (V)	16.4 ± 0.5 ^abc^	10.1 ± 1.2 ^bcd^	4.2 ± 0.2 ^abc^	20.7 ± 0.6 ^a^	0.75 ± 0.01 ^a^
Se (VI) + I (−I)	13.3 ± 0.8 ^bc^	8.14 ± 0.5 ^de^	3.3 ± 0.2 ^c^	16.1 ± 0.8 ^e^	0.76 ± 0.02 ^a^
Se (VI) + I (V)	16.4 ± 0.5 ^abc^	11.2 ± 0.3 ^bc^	4.3 ± 0.1 ^ab^	18.3 ± 0.3 ^bcd^	0.74 ± 0.01 ^a^

**Legend:** ETS, electron transport system; Se (IV), sodium selenite (Na_2_SeO_3_); Se (VI), sodium selenate (Na_2_SeO_4_); I(−I), potassium iodide (KI); I(V), potassium iodate (KIO_3_). Data are the means ±SE. Means followed by different superscript letters are significantly different at *p* < 0.05 (*n* = 5).

## Supplementary Materials

The following are available online at https://www.mdpi.com/2223-7747/9/12/1766/s1, Table S1: Weather conditions during the field experiment.

## References

[1] Schomburg L., Köhrle J. (2008). On the importance of selenium and iodine metabolism for thyroid hormone biosynthesis and human health. Mol. Nutr. Food Res..

[2] El-Ramady H., Abdalla N., Alshaal T., El-Henawy A., Faizy S.E.-D.A., Shams M.S., Domokos-Szabolcsy É. (2015). Selenium and Its Role in Higher Plants.

[3] Jerše A., Kacjan Maršić N., Kroflič A., Germ M., Šircelj H., Stibilj V. (2018). Is foliar enrichment of pea plants with iodine and selenium appropriate for production of functional food. Food Chem..

[4] Krzepiłko A., Prażak R., Skwaryło-Bednarz B., Molas J. (2019). Agronomic biofortifcation as a means of enriching plant foodstufs with iodine. Acta Agrobot..

[5] Allen L., de Benoist D., Dary O., Hurrell R. (2006). Guidelines on Food Fortification with Micronutrients Geneva: World Health Organization. http://www.who.int/iris/handle/10665/43412.

[6] Zimmermann M.B., Jooste P.L., Pandav C.S. (2008). Iodine-Deficiency disorders. Lancet.

[7] White P.J., Broadley M.R. (2009). Biofortification of crops with seven mineral elements often lacking in human diets—Iron, zinc, copper, calcium, magnesium, selenium and iodine. New Phytol..

[8] Gonzali S., Kiferle C., Perata P. (2017). Iodine biofortification of crops: Agronomic biofortification, metabolic engineering and iodine bioavailability. Curr. Opin. Biotechnol..

[9] Humphrey O.S., Young S.D., Bailey E.H., Crout N.M.J., Ander E.L., Hamilton E.M., Watts M.J. (2019). Iodine uptake, storage and translocation mechanisms in spinach (*Spinacia oleracea* L.). Environ. Geochem. Health.

[10] Brown K.M., Arthur J.R. (2001). Selenium, selenoproteins and human health: A review. Public Health Nutr..

[11] Hawrylak-Nowak B., Hasanuzzaman M., Matraszek-Gawron R., Hasanuzzaman M. (2018). Mechanisms of selenium-induced enhancement of abiotic stress tolerance in plants. Plant Nutrients and Abiotic Stress Tolerance.

[12] Rayman M.P. (2000). The importance of selenium to human health. Lancet.

[13] Zhu S.M., Liang Y.L., Gao D.K., An X.J., Kong F.C. (2017). Spraying foliar selenium fertilizer on quality of table grape (*Vitisvinifera* L.) from different source varieties. Sci. Hortic. Amst..

[14] Dall’Acqua S., Ertani A., Pilon-Smits E.A.H., Fabrega-Prats M., Schiavon M. (2019). Selenium biofortification differentially affects sulfur metabolism and accumulation of phytochemicals in two rocket species (*Eruca sativa* Mill. and *Diplotaxis tenuifolia*) grown in hydroponics. Plants.

[15] Li H.F., McGrath S.P., Zhao F.J. (2008). Selenium uptake, translocation and speciation in wheat supplied with selenate or selenite. New Phytol..

[16] Cartes P., Gianfreda L., Paredes C., Mora M.L. (2011). Selenium uptake and its antioxidant role in ryegrass cultivars as affected by selenite seed pelletization. J. Soil Sci. Plant Nutr..

[17] Zhu Y.G., Huang Y., Hu Y., Liu Y., Christie P. (2004). Interactions between selenium and iodine uptake by spinach (*Spinacia oleracea* L.) in solution culture. Plant Soil.

[18] Bieżanowska-Kopeć R., Pysz M., Kapusta-Duch J., Aneta K., Sylwester S., Koronowicz A., Piątkowska E., Rakoczy R., Skoczylas L., Leszczyńska T. (2016). The effects of peeling and cooking on the mineral content and antioxidant properties in carrots enriched with potassium iodate and/or selenite (SeIV) and selenite (SeVI). Int. J. Food Sci. Nutr..

[19] Smoleń S., Skoczylas Ł., Ledwożyw-Smoleń I., Rakoczy R., Kopeć A., Piątkowska E., Kapusta-Duch J. (2016). Biofortification of carrot (*Daucus carota* L.) with iodine and selenium in a field experiment. Front. Plant Sci..

[20] Smoleń S., Kowalska I., Sady W. (2014). Assessment of biofortification with iodine and selenium of lettuce cultivated in the NFT hydroponic system. Sci. Hortic. Amst..

[21] Jerše A., Kacjan Maršić N., Šircelj H., Germ M., Kroflič A., Stibilj V. (2017). Seed soaking in I and Se solutions increases concentrations of both elements and changes morphological and some physiological parameters of pea sprouts. Plant. Physiol. Biochem..

[22] Golubkina N., Kekina H., Caruso G. (2018). Yield, quality and antioxidant properties of indian mustard (*Brassica juncea* L.) in response to foliar biofortification with selenium and iodine. Plants.

[23] Germ M., Stibilj V., Šircelj H., Jerše A., Kroflič A., Golob A., Kacjan Maršić N. (2019). Biofortification of common buckwheat microgreens and seeds with different forms of selenium and iodine. J. Sci. Food Agric..

[24] Golob A., Novak T., Kacjan Maršić N., Šircelj H., Stibilj V., Jerše A., Kroflič A., Germ M. (2020). Biofortification with selenium and iodine changes morphological properties of *Brassica oleracea* L. var. gongylodes) and increases their contents in tubers. Plant Physiol. Biochem..

[25] Nwafor I.C., Shale K., Achilonu M.C. (2017). Chemical composition and nutritive benefits of chicory (*Cichorium intybus*) as an ideal complementary and/or alternative livestock feed supplement. Sci. World J..

[26] Rossetto M., Lante A., Vanzani P., Spettoli P., Scarpa M., Rigo A. (2005). Red chicories as potent scavengers of highly reactive radicals: A study on their phenolic composition and peroxyl radical trapping capacity and efficiency. J. Agric. Food Chem..

[27] Statistical Office of the Republic of Slovenia (2013). Statistični letopis Republike Slovenije. Statistical Yearbook of the Republic of Slovenia.

[28] Pelko N. (2020). Bilanca Proizvodnje in Potrošnje Zelenjave na leto in Kratko Razmišljanje Načrtovanju Pridelave Zelenjave in Krompirja v letu 2020. https://www.kgzs.si/uploads/slike/bilanca_proizvodnje_in_potrosnje_zelenjave_2_clanek_splet.pdf.

[29] Germ M., Stibilj V., Osvald J., Kreft I. (2007). Effect of selenium foliar application on chicory (*Cichorium intybus* L). J. Agric. Food Chem..

[30] Blasco B., Rios J.J., Leyva R., Melgarejo R., Constan-Aguilar C., Sanchez-Rodriguez E., Rubio-Wilhelmi M.M., Romero L., Ruiz J.M. (2011). Photosynthesis and metabolism of sugars from lettuce plants (*Lactuca sativa* L. var. longifolia) subjected to biofortification with iodine. Plant Growth Regul..

[31] Golob A., Kroflič A., Jerše A., Kacjan Maršić N., Šircelj H., Stibilj V., Germ M. (2020). Response of pumpkin to different concentrations and forms of selenium and iodine, and their combinations. Plants.

[32] Kopsell D.A., Sams C.E., Barickman T., Deyton D.E., Kopsell D.E. (2009). Selenization of basil and cilantro through foliar applications of selenate-selenium and selenite-selenium. Hortic. Sci. Hortic..

[33] Kacjan Maršič N., Golob A., Šircelj H., Mihorič M., Kroflič A., Stibilj V., Germ M. (2020). Effects of exogenous selenium in different concentrations and forms on selenium accumulation and growth of Spinach (*Spinacia oleracea* L.). J. Agric. Sci. Technol. Iran.

[34] Terry N., Zayed A.M., de Souza M.P., Tarun A.S. (2000). Selenium in higher plants. Annu. Rev. Plant Physiol. Plant. Mol. Biol..

[35] Smoleń S., Skoczylas Ł., Ledwożyw-Smoleń I., Rakoczy R., Kopeć A., Piątkowska E., Bieżanowska-Kopeć R., Pysz M., Koronowicz A., Kapusta-Duch J. (2016). Iodine and selenium biofortification of lettuce (*Lactuca sativa* L.) by soil fertilization with various compounds of these elements. Acta Sci. Pol. Hortorum Cultus.

[36] Packard T.T., Jannasch H.W., Williams P.J. (1985). Measurement of electron transport activity of microplankton. Advances in Aquatic Microbiology.

[37] Gaberščik A., Germ M., Škof A., Drmaž D., Trošt Sedej T. (2002). UV-B radiation screen and respiratory potential in two aquatic primary producers: *Scenedesmus quadricauda* and *Ceratophyllum demersum*. Verhandlungen.

[38] Bartoli C.G., Gomez F., Gergoff G., Guiam J.J., Puntarulo S. (2005). Up-regulation of the mitochondrial alternative oxidase pathway enhances photosynthetic electron transport under drought conditions. J. Exp. Bot..

[39] Kato S., Wachi T., Yoshihira K., Nakagawa T., Ishikawa A., Takagi D., Tezuka A., Yoshida H., Yoshida S., Sekimoto H. (2013). Rice (*Oryza sativa* L.) roots have iodate reduction activity in response to iodine. Front. Plant Sci..

[40] Schreiber U., Kohl M., Klimant I., Reising H. (1996). Measurement of chlorophyll fluorescence within leaves using a modified PAM Fluorometer with a fiber-optic microprobe. Photosynth. Res..

[41] Ćustić M., Poljak M., Čoga L., Ćosić T., Toth N., Pecina M. (2003). The influence of organic and mineral fertilization on nutrient status, nitrate accumulation, and yield of head chicory. Plant Soil Environ..

[42] Bortolini L., Tolomio M. (2019). Influence of irrigation frequency on radicchio (*Cichorium Intybus* L.) yield. Water.

[43] https://www.gov.si/teme/integrirana-pridelava/19.11.2020.

[44] Lichtenthaler H.K., Buschmann C. (2001). Chlorophylls and carotenoids: Measurement and characterization by UV-VIS spectroscopy. Current Protocols in Food Analytical Chemistry.

[45] Lichtenthaler H.K., Buschmann C. (2001). Extraction of phtosynthetic tissues: Chlorophylls and carotenoids. Current Protocols in Food Analytical Chemistry.

[46] Kenner R.A., Ahmed S.I. (1975). Measurements of electron transport activities in marine phytoplankton. Mar. Biol..

[47] Packard T. (1971). The measurement of electron transport activity in marine phytoplankton. J. Mar. Res..

