# Supplementary material for: Significant Accumulation of Iodine and Selenium in Chicory (Cichorium intybus L. var. foliosum Hegi) Leaves after Foliar Spraying
Source: Plants (Basel). 2020 Dec 13;9(12):1766. doi: 10.3390/plants9121766 (PMC7764295; doi:10.3390/plants9121766)
Supplement: Supplementary file 1 [file plants-09-01766-s001.pdf]

Supplementary Information Table S1: Weather conditions during the field experiment.

LJUBLJANA - BEŽIGRAD lon=14.5124 lat=46.0655 altitude=299m	Mean day T [°C]	Mean wind [m/s]	Mean relative humidity [%]	Rainfall [mm]	Solar iradiation [h]
**2015-07-27**	18.4	0.9	87	0.8	0.2
**2015-07-28**	19.1	1.1	86	5.8	0
**2015-07-29**	17.9	1.6	86	3.2	0
**2015-07-30**	15.2	0.4	94	19.4	0
**2015-07-31**	18.1	1.4	74	25.8	8.1
**2015-08-01**	20	0.7	72	0	10.6
**2015-08-02**	21.1	1.4	78	6.2	6.2
**2015-08-03**	23.2	1.1	74	0.1	7.4
**2015-08-04**	24.9	1	70	0	11.2
**2015-08-05**	26.1	1.1	68	0	8.9
**2015-08-06**	26.7	1.1	67	0	12.7
**2015-08-07**	26.4	0.6	70	0	12.8
**2015-08-08**	26	1.9	64	0	10.2
**2015-08-09**	26.2	1.5	57	0	12.7
**2015-08-10**	24.9	1	58	0	12.7
**2015-08-11**	24.6	1	57	0	10.4
**2015-08-12**	25.2	0.6	61	0	11
**2015-08-13**	26.8	0.9	60	0	11
**2015-08-14**	25.2	0.8	70	0	10.3
**2015-08-15**	23	0	72	2	4.5
**2015-08-16**	20.8	0.6	79	2	4.5
**2015-08-17**	18.1	0.6	96	7.9	0
**2015-08-18**	19.9	0.7	76	3.9	5.7
**2015-08-19**	16.8	0.7	93	0.2	0
**2015-08-20**	19.1	1.5	79	33.5	3.5
**2015-08-21**	17.8	0.8	81	0	0.5
**2015-08-22**	17.2	0.9	71	0.2	9.3
**2015-08-23**	18.7	0.7	71	0	9
**2015-08-24**	18.3	0	82	0	0.6
**2015-08-25**	17.3	0.9	97	3.2	3.9
**2015-08-26**	19.3	0.7	75	37.2	6.4
**2015-08-27**	21.5	0.3	69	0	8.8
**2015-08-28**	23.1	1.8	69	0	12.2
**2015-08-29**	23.7	0.7	72	0	11.1
**2015-08-30**	24.7	0.5	74	0	10.2
**2015-08-31**	24.2	1.8	66	0	11.7
**2015-09-01**	24.3	1.3	60	0	11.5
**2015-09-02**	20.3	0.1	79	0	5.9
**2015-09-03**	20.2	0.1	79	0.7	2.9
**2015-09-04**	17.5	0.1	90	21.4	1
**2015-09-05**	14.1	1.9	95	45.6	0
**2015-09-06**	15.1	0.2	74	19	8.6
**2015-09-07**	13.9	0.2	67	0.3	11.2
**2015-09-08**	14.8	0.3	70	0	11.2
**2015-09-09**	14.1	1.1	72	0	10.4
**2015-09-10**	14.6	0.9	71	0	5.6

LJUBLJANA - BEŽIGRAD lon=14.5124 lat=46.0655 altitude=299m	Mean day T [°C]	Mean wind [m/s]	Mean relative humidity [%]	Rainfall [mm]	Solar iradiation [h]
**2015-09-11**	15.1	0.7	76	0	6.6
**2015-09-12**	17.4	1.7	76	0	9
**2015-09-13**	19.1	0.3	68	0	6.5
**2015-09-14**	18.6	0.1	85	0	3.9
**2015-09-15**	20.6	2.1	71	1.4	8.6
**2015-09-16**	20.5	0.9	88	0	3.8
**2015-09-17**	22.6	1.7	77	6.8	7.7
**2015-09-18**	22.8	1	77	0	8.1
**2015-09-19**	18.8	1.8	80	0.2	0.3
**2015-09-20**	18.2	1	66	0.8	4.1
**2015-09-21**	14.1	0.9	69	0	10.4
**2015-09-22**	14.4	0.4	72	0	9.6
**2015-09-23**	13.6	2	85	0	3.2
**2015-09-24**	12.1	0.4	93	36.7	0
**2015-09-25**	13.6	0.7	95	6.9	0
**2015-09-26**	15.3	0.6	78	11.9	1.4
**2015-09-27**	14.3	1.9	65	0	0
**2015-09-28**	11.8	2.7	67	0	5.6
**2015-09-29**	12.5	2.3	60	0	3.9
**2015-09-30**	11	2.3	71	0	2.2
**2015-10-01**	12.1	1.8	67	0.1	0.3
**2015-10-02**	13.2	1.3	76	0	0.9
**2015-10-03**	13.2	0.9	88	0	3
**2015-10-04**	14.6	1.4	86	4.1	0.2
**2015-10-05**	14.5	0.5	85	3.8	6.9
**2015-10-06**	13.6	0.5	95	0	0
**2015-10-07**	14.7	0.8	96	18.1	0
**2015-10-08**	14.3	0.2	90	14.2	0.3
**2015-10-09**	14.3	0.7	86	0.8	0
**2015-10-10**	11.9	2.1	90	0	0
**2015-10-11**	10.9	1.2	94	8.3	0
**2015-10-12**	9.9	1	85	4.5	0
**2015-10-13**	9.4	0.5	95	1.4	0
**2015-10-14**	9.6	0.8	98	13.8	0
**2015-10-15**	10.7	0.7	97	25.8	0
**2015-10-16**	9.7	1.3	88	13.9	2.4
**2015-10-17**	10.1	0.5	84	6.6	5.5
**2015-10-18**	12.1	2	75	0	6.7
**2015-10-19**	8.4	1.1	92	7.2	0
**2015-10-20**	8.7	0.6	82	18	6.2
**2015-10-21**	7.9	1.1	83	0	6.1

Source: https://meteo.arso.gov.si/met/sl/archive/
